# Estimate of within population incremental selection through branch imbalance in lineage trees

**DOI:** 10.1093/nar/gkv1198

**Published:** 2015-11-19

**Authors:** Gilad Liberman, Jennifer I.C. Benichou, Yaakov Maman, Jacob Glanville, Idan Alter, Yoram Louzoun

**Affiliations:** 1 Gonda Multidisciplinary Brain Research Center, Bar Ilan University, Ramat-Gan 5290002, Israel; 2 Department of Mathematics, Bar Ilan University, Ramat-Gan 5290002, Israel; 3 Department of Immunobiology, Yale University School of Medicine, New Haven, CT 06520-8011, USA; 4 Howard Hughes Medical Institute, New Haven, CT 06519, USA; 5 Program in Computational and Systems Immunology, Stanford University, Stanford, CA 94305, USA; 6 Department of Pathology, Transplantation and Infection, Stanford University, Stanford, CA 94305, USA; 7 Program in Immunology, Transplantation and Infection, Stanford University, Stanford, CA 94305, USA; 8 Distributed Bio, San Francisco, CA 94080, USA

## Abstract

Incremental selection within a population, defined as limited fitness changes following mutation, is an important aspect of many evolutionary processes. Strongly advantageous or deleterious mutations are detected using the synonymous to non-synonymous mutations ratio. However, there are currently no precise methods to estimate incremental selection. We here provide for the first time such a detailed method and show its precision in multiple cases of micro-evolution. The proposed method is a novel mixed lineage tree/sequence based method to detect within population selection as defined by the effect of mutations on the average number of offspring. Specifically, we propose to measure the log of the ratio between the number of leaves in lineage trees branches following synonymous and non-synonymous mutations. The method requires a high enough number of sequences, and a large enough number of independent mutations. It assumes that all mutations are independent events. It does not require of a baseline model and is practically not affected by sampling biases. We show the method's wide applicability by testing it on multiple cases of micro-evolution. We show that it can detect genes and inter-genic regions using the selection rate and detect selection pressures in viral proteins and in the immune response to pathogens.

## INTRODUCTION

The phenotypic effect of genotypic changes and whether these changes affect the function and the fitness of the organism remain one of the most basic questions in many biological settings. Mutations can affect the average offspring number of an organism. An increase in the number of offspring is often treated as an indicator for a better fitness and vice versa. Given an observed set of genes within a population, a central question arising in many domains of population dynamics is whether the observed genetic constitution of a population can be explained by a neutral random drift, or whether one must incorporate the effect of mutations on the fitness to explain the observed distribution of genes in the population.

This question is asked at the general level in evolution, where a debate has emerged between selection-based evolution and neutral evolution (1–3). It is also often addressed at the micro-evolution level, as happens for example in viral escape mutations to avoid immune mediated destruction (4–6), the dynamics of specific clones in the B cell response against pathogens (7,8) or maternal inheritance within a population (9,10). These cases are examples of processes involving rapid asexual reproduction, where constant diversification and possibly adaptation occur with a high mutation rate.

When the effect of mutations is drastic, as is the case for strongly deleterious or advantageous mutations, a clear genetic signature of the selection can be observed in nucleotide composition, and multiple methods have been proposed for measuring selection in such cases. Some of these measures rely on the ratio of synonymous (S) to non-synonymous (NS) mutations. Specifically, a comparison of the observed and expected NS/(NS+S) ratios is often used as a measure for selection. The expected ratio is calculated based on an underlying mutation probability model (e.g. (11–13)), or on genetic regions where no selection is assumed to occur (14). An increased frequency of NS mutations is an indication for positive selection and vice versa. These methods are often useful, when a good estimate of the baseline mutation model is available. They may however lead to erroneous conclusions when the baseline mutation model (i.e. the expected probability of each mutation type) is inaccurate, as happens for example in immunoglobulin sequences (15).

In many cases of micro-evolution, the observed time scale of the dynamics is limited, and the fitness (dis)advantage induced by mutations may be limited. In such a case, the fixation probability is low, and S to NS based methods is less useful. A different approach proposed for detecting weak selection is to use properties of lineage trees. Two of the most powerful such measures proposed for the detection of selection (16,17) are Sackin's and Colless's statistics (18–21). Sackin's index is the average root-leaf distance (over all leaves). Colless's index is the sum of imbalance over all nodes, where a node's imbalance is taken to be the difference in number of leaves between the bigger and smaller sub-trees. These measures are tested versus a neutral model, which is usually the Yule model, where a tree is constructed by giving each branch the same probability to split (22). Other statistics do not use trees but are based on the number of segregating sites, most notably Tajima's D (23).

These methods have two well-known limitations. They do not distinguish between S and NS mutations and statistical power is lost. Most of these methods measure deviation from a neutral model and cannot differ between different types of selection, e.g. positive and negative ones.

We here offer a more direct approach to measure incremental selection within species passing a continuous adaptation, which is directly related to a quantitative definition of incremental selection. This new method overcomes limitations of the S to NS mutation ratio and of the tree shape based selection detection methods, by accounting for the completing information found in each of the two, that is, the classification into mutation types, and the imbalance between different sub-trees.

As is the case for any method, this method requires enough observations to be valid. Specifically, the total number of independent mutations should be of the order of at least 10–20 per genetic region of interest. In parallel, the total number of the leaves in the phylogeny constructed should be high enough to obtain a large enough difference between branches, which requires at least a hundred sequences. Finally the population dynamics should not be dominated by strongly advantageous mutations that would fixate rapidly and erase the history of all other mutations.

## MATERIALS AND METHODS

### Alignment and phylogenetic trees

The DNA sequences of different viruses were aligned using TranslatorX (24), which aligns nucleotide sequences based on their corresponding amino acid translations. Lineage trees were then produced from the aligned sequences using Maximum Parsimony (Phylip bioinformatics tool package-version 3.69) (25). For the mouse data, three other tree construction techniques: Neighbor Joining (NJ), Maximum Likelihood (ML) and UPGMA (Unweighted Pair Group Method with Arithmetic Mean) were used to validate robustness to construction algorithm. For samples with over 100 sequences, a NJ algorithm was used in the same package. For each group of sequences, a genetically distant ‘outgroup’ sequence was added to position the root of the tree, and reconstruct the ancestral sequences. To avoid ambiguous nucleotides in internal nodes, when both child sequences had a gap in a certain locus, the parental nucleotide was changed to a gap as well. If one of the child sequences had a non-ambiguous nucleotide, the parental nucleotide was changed accordingly.

While recombination may be important in general in viruses (26), we have ignored its effect, and did not find evidences for it in our current data set. In the mitochondrial data set and the Ig data sets, a single lineage tree was built for each group of sequences. The separation into regions was performed after the construction of the lineage tree.

### Selection score

**Figure 1.**
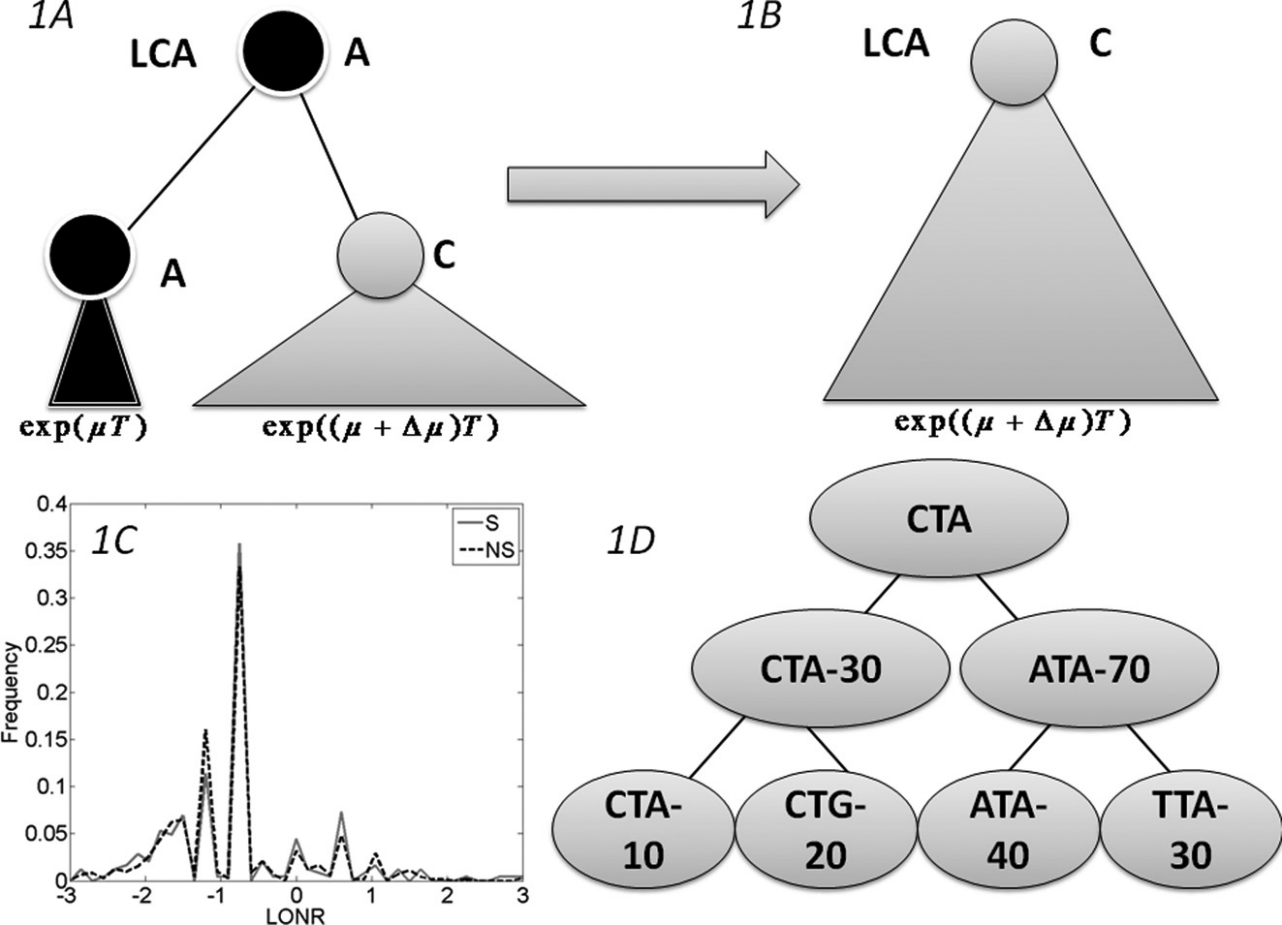
The branch imbalance framework and examples. (**A**) Schematic view of a branch corresponding to a mutation event. Following a mutation, the population can be expanded (or reduced), the advantage will lead to an exponentially growing difference in the number of offspring in parallel branches descending from the same internal origin. (**B**) After some time, one branch will take over the entire sample, and the information carried in the ratio between the branches will be lost. (**C**) LONR values histogram for one simulated sequence pool, simulated under naive multiplication from unique ancestral sequence. While the average is not 0, there is no difference between branches following S and NS mutations. (**D**) Example of a tree. In the left branch a mutation occurred from CTA to CTG, and the ratio between the mutated and un-mutated branches number of offspring is 20/10. In the right branch, a mutation from ATA to TTA occurred, with a ratio of 30/40. In the root, a mutation from CTA to ATA occurred with a ratio of 70/30.

Given a tree, each mutation event was assigned: (i) an NS or a S mutation flag by its effect on the amino-acid translation of the containing codon; (ii) the location of the mutation (related gene where applicable, and number of nucleotides from the beginning of the sequence, otherwise); and (iii) The log of the ratio between the number of leaves (sequences) in the sub-tree following the mutation branch and the number of leaves in the sub-tree following the non-mutated branch (see Figure 1, and Supplementary Figures S1 and S2). This ratio is denoted the Log Offspring Number Ratio (LONR). This log-ratio is positive if the number of final sequences marked by the tree construction algorithm as descendants of the mutated sequence is larger than the number of final sequences marked as descendants of the non-mutated sequence, suggesting some better fitness of the mutated sequence, or positive selection, and negative in the opposite case. For each area of the sequence, a *t*-test is performed (unpaired, unequal variances) between the NS and S mutations.

When distant alleles are combined into the same lineage tree, long branches with a large number of mutations will emerge. If the alleles have unequal sampling depth, the LONR of mutations along these branches will deviate from 0. In order to avoid such a bias, we cut branches with length above some threshold. In such a case, the tree is divided into sub trees, and the LONR is computed for each subtree by itself. LONR values are not computed from mutations in the removed branches.

Another effect that can bias the LONR score is the effect of mutations in lower nodes on internal nodes above them. For example, assume that internal node x branches into sub trees X1 and X2, with X2 containing much more leaves than X1. Let us denote the subtrees of X2 by X2a and X2b. The large number of leaves in X2 could be the result of a highly advantageous mutations in X2a. In order to test for such cases, we check what would happen to the LONR score if the number of leaves in X2a would be replaced by the number of leaves in X2b. Specifically, we checked if the LONR value between X1 and X2 would switch signs if X2 = X2a+X2b is replaced by X2 = 2*min(X2a,X2b) (i.e. X2a+X2a in this case). If the sign of the LONR changes signs in such a case, we flag the LONR value of the mutations between X2 and X1. We then let the user decide whether to incorporate this mutation into the analysis.

### Simulation

A sequence pool simulating neutral evolution was generated from a random original sequence of 348 nucleotides, with a constant multiplication rate of two offspring per organism. Two equal size regions (174 nt. each) were defined with uniform mutation probabilities with average mutation rate of 1/2 and 1 mutations per generation. The population was sampled in different sample sizes and along different generations. In each sampling, one of the eight first siblings (the third generation) was chosen randomly, and its descendants had a twice higher probability of being sampled, effectively simulating sampling bias for a specific clone. The process was repeated 1000 times. We tracked all sequences in the simulation, and the last generation of the simulation was sampled to produce the lineage trees, using the algorithms mentioned above.

A second simulation framework was used to test more complex aspects of selection. A population was initiated with a set of sequences. Each sequence was associated with an initial equal fitness. S mutations did not affect the fitness, while NS mutations could multiply the fitness by a factor lambda. This factor was not uniform along the sequence and was larger than 1 in some positions along the sequence and lower than 1 in other positions. The per-sequence factor was the product of the factors of all its NS mutations. At each division cycle, each sequence produced a random number of offspring following a Poisson distribution with an average equal to its fitness. If following the division cycle, the total number of sequences passed 10 000, random sequences were removed to reach this number.

We used the same simulation to compare the LONR score of real and reproduced trees. We kept the sequences of all intermediate stages in order to reconstruct the lineage tree in different generations. We then computed the LONR score difference between S and NS mutations based on the real trees and the reconstructed trees.

In order to further test our results, a coalescent simulation was used. We simulated the ancestry of a sample of sequences following the evolution back in time of Kingman's coalescent. At every time step until the Time to Most Recent Common Ancestor (TMRCA), every pair of sequences in the current population has an equal chance to coalesce into an ancestral sequences, which replaces both of them in the population. After the ancestry tree has been generated, we superimposed on it a mutation model—every node undergoes a single nucleotide polymorphism mutation at an equal probability, where the mutation itself is chosen randomly using either the Juke–Cantor or the Kimura mutation models. The resulting sequences where then used as an input for the LONR method. Note that the LONR analysis used the NJ-constructed tree, and not the real tree emerging from the coalescent.

### Statistical analysis

For the mitochondrial sequences, the analysis was performed using a sliding window of 400 nucleotides, shifted by steps of 20 nt. The *P*-values are presented for each window, along with the differences in the mean LONR values between the NS and S mutations. In order to asses areas where selection forces are presented for NS and S events alike, a one sample *t*-test is performed on LONR values of all mutation events (i.e. NS and S mutations). When reporting the final results, a FDR correction was performed to account for the large number of windows.

For the viral sequences, a single tree was constructed for each virus from the obtained sequences, and a two-way ANOVA test was performed for assessing the significance of the NS versus S grouping, the epitopes versus non-epitopes grouping and interactions between the two.

For the transgenic mice data, trees were constructed for different clones and LONR values were collected from all trees, grouped by the two mouse types. Mean NS–S LONR values are reported along with two-sample *t*-test *P*-values.

For the immunoglobulin data, the receptors where clustered by isotype (IgA and IgG). Lineage trees were constructed and the sequences were divided to CDR and FWR regions. Mean LONR NS–S difference was computed per clone and per region along with two sample *t*-test *P*-values.

### Viral and mitochondrial sequences

All sequences were obtained from the NCBI nucleotide database (27). We have used sequences from Influenza A (1000 sequences for segment 1 to segment 6), HBV (1694, 2370, 211 and 999 sequences for Core, Polymerase, Surface and X, accordingly), HIV (179, 823, 731, 159, 757 and 150 for Env, Gag, Pol, Rev, Tat and Vpu accordingly) and HPV (105, 89, 88, 72 and 121 for E2, E6, E7, L1 and L2, accordingly). For the sake of lineage trees design (see the next section), we have defined an outgroup for each set using genetically distant homologues (e.g. Influenza B for Influenza trees).

The human mitochondrial sequences were all of the full genome nucleotide sequences available at the NCBI, with a length of at least 15574 and at most 16581 nucleotides. 2689 sequences were used with hosts from multiple regions including large cohorts from China and India.

### Mouse data

The sequences from transgenic mice were obtained from two H chain transgenic mice (28,29) that were backcrossed with Jh KO/Balb mice (29,30) for nine or more generations. All mice were maintained under specific pathogen-free conditions and sacrificed at 6–10 week of age. Mice were immunized i.p. with 50μg of NP25-chicken gamma-globulin (CGG) precipitated in alum or precipitated alum alone as a control. B cells were sequenced from micro-dissections in germinal centers of these mice, 16 days after the immunization. One mice type had an initial low affinity for the antigen, while the other had an initial high affinity.

### Immunoglobulin sequences

Over 500 000 B cell receptors were sampled from each donor in 12 donors, using 454 sequencing and a RACE protocol. The details of the sequencing and the validity checks were previously described (31). For each sequence, the most fitting V, J and V-J distance was found by maximizing the relative number of non-mutations for both V and J segments. The sequences were then clustered according to the most fitting V and J as well as the distance between V and J, and were truncated to 159 nucleotides from the end of the germline V and 20 nucleotides from the beginning of the germline J.

### Defining epitope regions

Epitopes were computed using three algorithms: a proteasomal cleavage algorithm (32), a transporter associated with antigen processing (TAP) binding algorithm (33), and the MLVO major histocompatibility complex (MHC) binding algorithm (34). We have computed epitopes for the 39 most common human leukocyte antigen (HLA) alleles and weighted the results according to the allele frequency in the global human population. The algorithms’ quality was systematically validated versus epitope databases and was found to induce low false positive (FP) and false negative (FN) error rates. These algorithms were validated in multiple previous analyses (35–42).

Each ninemer, in each aligned sequence, was scored according to the weighted frequency of alleles to which it binds. For longer sequences, each position in the sequence was scored according to the maximal score given to any ninemer containing it. For the whole aligned sequence population, these values were averaged on a per sequence manner, resulting in an epitope score per position. The positions that scored in the higher 15% were defined to be epitope related areas.

## RESULTS

### Selection

Assume a population originating from a single founder through division, with a given ancestral sequence in the genetic region of interest (a gene, a combination of genes or even a part of a gene). Mutations in this region can potentially affect the population dynamics. In such a case we would define positive selection as an increased average division/birth rate or a decreased average death rate following mutations (note that these are not precisely the same (43), but the distinction is beyond the scope of the current analysis). Similarly, a decrease in the division rate would be defined as negative selection. Obviously, each mutation by itself can have a positive, null or negative effect, but the definition of selection is based on the average population dynamics and not on the dynamics following a single mutation.

Let us follow a mutation that occurs within a population, if this mutation raises the average number of offspring per generation from $\mu$ to $\mu + \Delta \mu$, then by a time proportional to $\log ({\rm Population}\;{\rm size})/\Delta \mu$, the advantageous mutation will take over the population (44), and when the population is compared to its latest common ancestor (LCA), there is no direct evidence that such a mutation has occurred (Figure 1A, B). In this case the genetic composition of the population would be equivalent to the one expected in a neutral model. The only difference would be the addition of an NS mutation to the region of interest in the entire population. If an external reference (e.g. from the comparison to orthologues) that can help us define the sequence seeding the population is available, it can be used to infer that selection has taken place.

However, in many cases, evolution occurs over an intermediate period and is weak, leading to the coexistence of the two alleles (the mutated and the un-mutated one).In such cases, we expect the ratio between the two allele frequencies to be proportional to $e^{\Delta \mu T}$, where T is the time from the mutation to the sampling time (Figure 1A). For a single mutation, it is impossible to differentiate between the effect of selection and a non-uniform sampling where one branch is sampled more deeply. However, if multiple mutations occur in the genetic region of interest, and if mutations in this region increase the average number of offspring, we expect, on average, more offspring in branches that follow a mutation in this region than in branches emerging from the same direct ancestor with no mutations, and the opposite in the case of negative selection.

We thus propose to detect incremental selection using this imbalance in cases where most mutations are neither strongly deleterious nor strongly advantageous. Such cases are far from being rare and become more and more frequent as the depth of genetic sampling increases in many domains (45–49).

### Effect of sampling

Assume a sample from a population dynamics process, with a ‘real lineage tree’ representing the actual division and mutation process. In the real tree, the average ratio between the number of leaves under an internal node that has a given mutation and the parallel descendent of their common direct ancestor that does not have a mutation (i.e. its un-mutated sibling) should be 1. The same cannot be said of the reconstructed lineage tree based on the sampled distribution, following biases induced by the sampling or the tree construction algorithm (Figure 1C). More specifically, a branch with a specific mutation is one possible offspring out of many. Thus, this specific branch may be smaller than the parallel branch holding the other offspring. However, in the absence of selection, the ratio between the total number of offspring of a branch with a mutation and without one should be similar following S and NS mutations. Thus, the in order to estimate the presence of selection, one can simply compare this ratio (that we denote as the branch size imbalance) following S and NS mutations. NS and S mutations are defined here relative to their direct ancestor.

### LONR

We define a measure of selection induced by a mutation as the ratio of the sub tree size under a branch where the mutation occurred and the sub tree size in its direct sibling where no such mutation occurred. As mentioned above, such a measure by itself could be biased. We thus require a baseline to estimate the expected deviation of this ratio from one. A simple baseline is the observed ratio in S mutations, assumed not to pass selection.

To estimate selection in a genetic region, we thus compare the distribution of these ratios (more precisely the log of the ratios) in all S and NS mutations occurring in this region. If the mean of these ratios following S and NS mutations differ significantly, we argue that selection is taking place (Figure 1D).

Specifically, for each mutation occurring in one descendent of an internal node and not in the other, we compute the sub-tree size under the descendent with a mutation and the sub-tree under the descendent without a mutation. Positions where a mutation occurred in the two descendants (e.g. A- > C and A- > G) are ignored. The log of the ratio between the leaf numbers in these two sub-trees is defined as the Log Offspring Number Ratio (LONR) of this mutation. We then compute the LONR value for all S and NS mutations in the tree, and compare the S and NS LONR distributions (Supplementary Figures S1 and S2). These mutations are computed on the reproduced lineage tree, which may differ from the real tree. The effect of the tree production method will be further discussed.

The mutations of interest can be all the mutations occurring in a gene, a gene combination, or even a genetic region composing a part of a gene that can be continuous or discontinuous. Formally, we define a set of positions in a genetic segment, and only count mutations in this region (Supplementary Figures S1 and S2).

Note that this analysis is not sensitive to the details of the baseline model for the probability of either S or NS mutations, since their absolute number is never used in the analysis. The only case where such a model would affect the current measurement is in the extreme case that the baseline mutation probability would induce a much higher S than NS probability.

When one cannot separate S and NS mutations (e.g. in non-coding regions), a different baseline is required. A simple baseline to use could be the average LONR score along the genome of an organism, assuming that selection is limited to some regions. This is much less precise and more arbitrary than comparing S and NS LONR scores. However, it allows for a comparison of the effect of mutations in different parts of the genome.

### Simulated data

**Figure 2.**
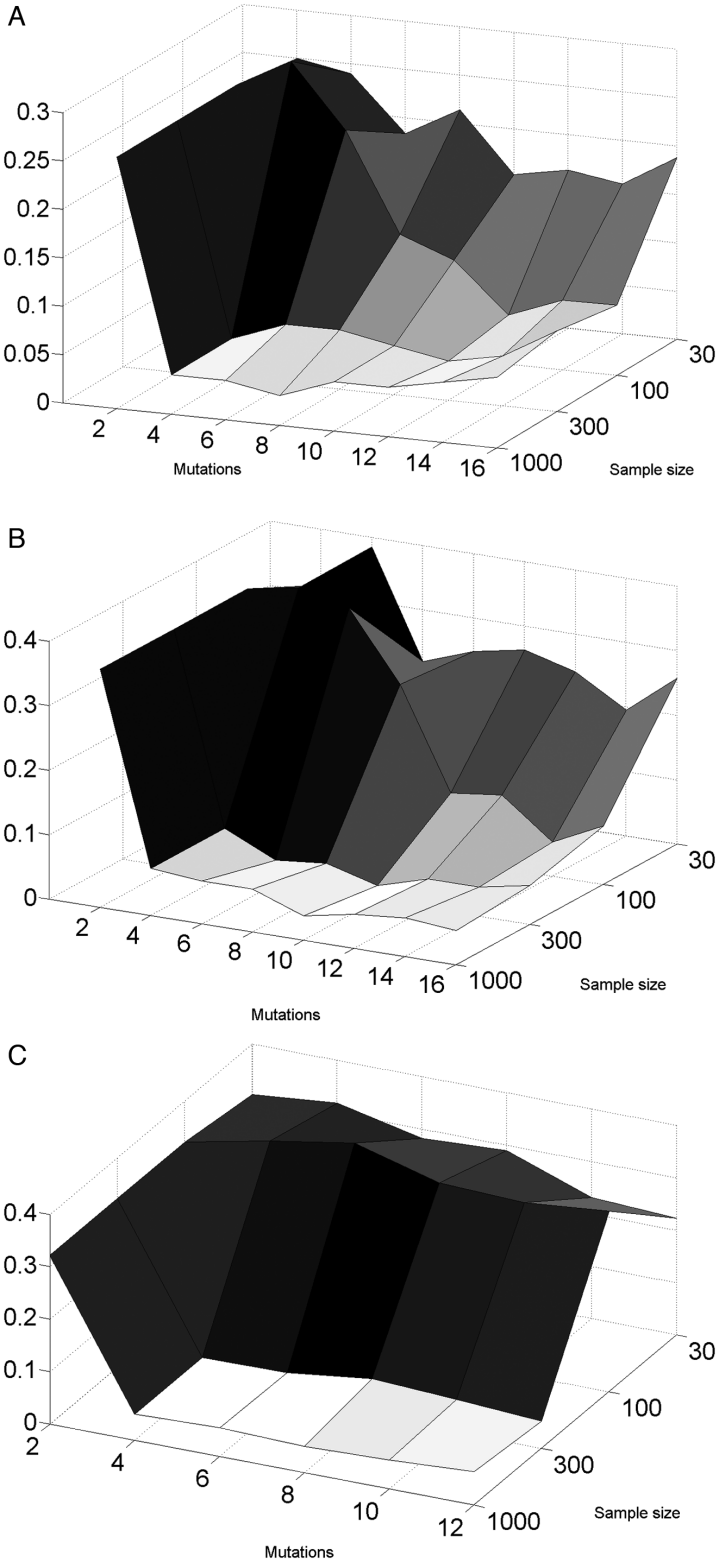
Fraction of lineage trees where selection was detected at the *P* = 0.05 level, as a function of the average number of mutations per sequence and the sample size. In all cases, the false positive fraction is around 0.05 (as expected randomly), when the sample size is above 300, and when there are at least 4–5 mutations per sequence. The results are consistent for (**A**) a uniform mutation rate (along the sequence), (**B**) non-uniform mutation rate, with some regions having a twice higher mutation rate, as well as when the mutation rate is non-uniform and the sampling is non uniform (**C**).

In order to check that the LONR does not detect selection in its absence, we simulated a Yule process, sampled the resulting sequences (see Materials and Methods for details), produced lineage trees and compared the LONR distribution following S and NS mutations (Figure 2A). In the regime of over 10–20 mutations per sequence and at least 300 sequences per tree, the False Positive (FP) rates (the cases where the LONR average is significantly different following S and NS mutations with *p*0.05) are near the expected 5% (Figure 2A). This range of mutation and sequence numbers is typical to most current applications of lineage trees and phylogenetics. We here limit the analysis to this range.

We have repeated the analysis with non-uniform mutation rates (position dependent mutation rates) and with sampling biases, and obtained similar results, as long as the S and NS mutation rates are of the same order of magnitude (Figure 2B and C). Specifically, sampling bias was simulated by oversampling descendants from one of the clones of the third generation. Again, in the domain of 300 sequences or more and an average of 10 mutations per sequence or more, the sampling effect and the non-uniform mutation rates along the sequence did not increase the error rate (see Materials and Methods for mutation and sampling models).

We avoid a major sampling bias by averaging over mutation events and not over sequences. Suppose for example, we would analyze a clone that led to two populations, one much more sampled than the other. This would affect a single internal node (probably the root), but the imbalance in all the other nodes would be unaffected. Within this internal node, the effect of over-sampling would be similar in S and NS mutations. Sub-sampling would have a significant effect only through the combined second-order effect of the sub-sampling in one node combined with the difference in the S and NS mutation frequencies. This effect is of no practical importance in all the examples studied here and probably in most realistic situations.

### Effect of contrasting selection in different regions and of different alleles

**Figure 3.**
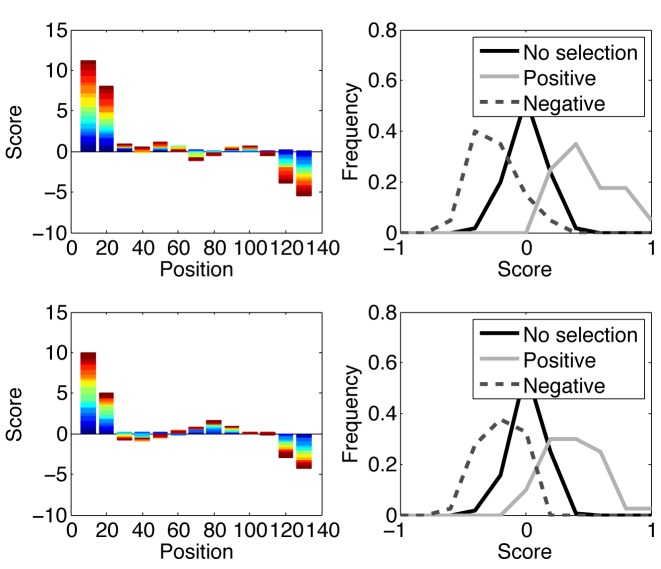
Average LONR score per region. Positive selection was simulated in the first 20 amino acids (60 nt), and negative selection was simulated for the last 20 AA in positions 121–140. In between, 100 AA were not subject to selection. We produced samples from the dynamics, and computed the average difference between the LONR score of S and NS mutations. In the left plots, Each color represent a different realization of the simulation, and each bar represents mutations occurring in a 20 AA sliding window. The *x*-axis is the center of each sliding window and the *y*-axis is the cumulative difference between the LONR of S and NS mutations over all realizations. The first and last columns represent regions inducing positive/negative selection. The second and one before last regions are mixed (half a window with selection and half without selection). All other windows do not contain positions affected by selection. The upper plots are initialized with a single sequence, while the two lower ones are initialized with two different alleles, one starting with a population twice larger than the other. The distance between the two initial alleles was 58 mutations (over 140*3 = 420 nt). The right wing plots represent the histograms of the values presented in the left plots, where positive selection are the two leftmost sliding windows, negative selection are the two rightmost sliding windows, and all other windows are defined to be no-selection.

The model underlying the analysis above is based on the assumption that NS mutations in some regions increase the fitness of the organism, while NS mutations in other regions decrease the fitness of the organism. The fitness of the organism is defined as the expected number of offspring per reproduction/division cycle. In order to test that the score above can detect selection in such a model, we simulated sequences that produce a random number of new offspring at each generation, with a constant mutation rate. Following each NS mutation, the expected number of offspring increases or decreases based on the region where the mutation occurred. We simulated multiple scenarios with combination of strong/weak positive and negative selection (either multiplying or dividing the affinity by 1.5/3). In all simulations, we defined in each genetic regions negative and positive selection and computed the LONR in each region. In all cases studied, no selection was detected by the LONR score in regions where no selection was simulated (Specifically 5% of tests showed selection at the 0.05 confidence level in regions where no selection was simulated). In regions where positive or negative selection was simulated, selection was detected in around 50% of the cases (Figure 3A and B). The presence of negative selection in one region did not affect the detection of positive selection in other regions and vice versa.

An alternative simulation framework is to combine a coalescent with a mutation model. We have further checked that the LONR method does not detect selection in its absence using such a model (Supplementary Figure S3 upper plot). As was the case for the forward simulation, no selection was detected by the LONR in the neutral model. We have repeated the analysis with non-uniform sampling by sampling only half the leaves in some of the branches. Again no selection was detected in the neutral model (Supplementary Figure S3 lower plot).

### Mitochondrial sequences

**Figure 4.**
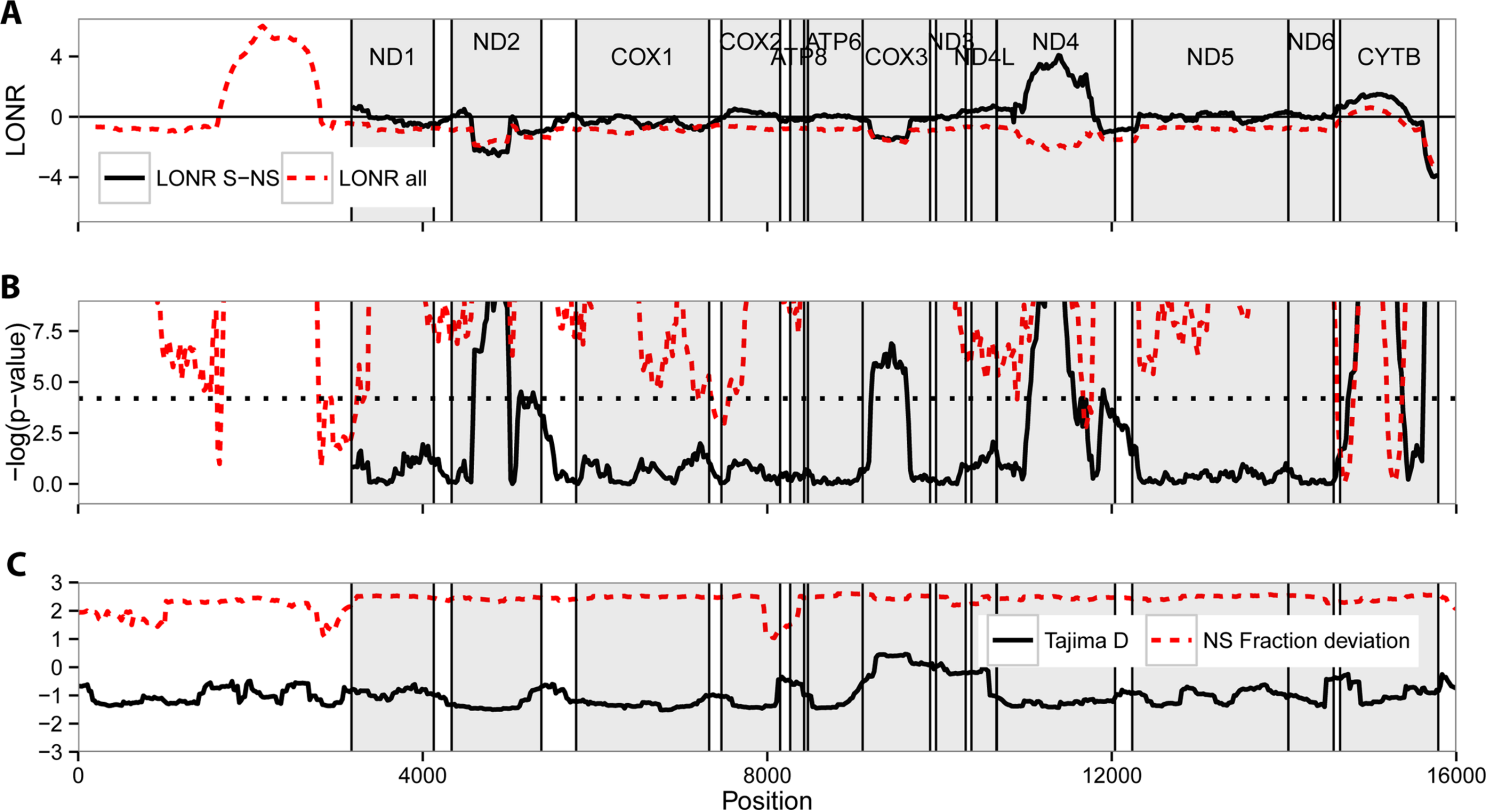
Mutation and selection pressure in full mitochondrial genomes. In all subplots the gray rectangles represent the Mitochondrial proteins. (**A**) Mean LONR values. The dashed red line is the LONR using all mutations with no base line. The dark line is the LONR when S to NS mutations are compared. (**B**) The –log(*P*-value) of one-sample *t*-test for divergence from the overall mean, and (dashed red line) and –log(*P*-value) of two-sample *t*-test for difference between NS and S mutation events (solid black line) as presented in the upper subplot. The dotted line refers to the Bonferroni correction threshold. The data were processed using sliding-window scheme with bin size of 400 nucleotides and 95% overlap. The gray rectangles represent again the mitochondrial genes. One can clearly see that the bands of selection follow closely the positions of some of the genes. The selection bands are narrower than the genes, following the effect of the sliding window. (**C**)The Tajima's D index and the NS/(NS+S)-NS0/(NS0+S0) index. The two indices do not detect selection in the ribosomal RNA and are not sensitive to the precise positions of genes for most genes.

A typical case where the number of generations is low and the mutation rate is high is maternal inheritance in the human mitochondrial genome. We sampled 3,106 sequences from published mitochondrial genomes that passed our quality validation checks (see Materials and Methods). We computed the average LONR value over all positions using a sliding window of 400 nucleotides and 95% overlap between windows (i.e. each window starts 20 nt. after the previous window). Not all the mitochondrial genome is coding. We have thus first tested the distribution of the LONR score for all mutations. This distribution is non-uniform with very large peaks (Figure 4A). As mentioned above, a baseline is required for the estimate of the significance of the results. We have used zero as a baseline, and estimated the significance of the LONR score using a one sample *t*-test with FDR correction (Figure 4B). The observed peaks overlap with the known mitochondrial genes as well as an rRNA region in positions 1671–3229 (Figure 4A and B). In other words, the LONR delineates important regions in the mitochondrial genomes, where mutations have an important effect, with no a-priori knowledge. Specifically, the strongest positive selection force is in the area between 1671 and 3229 nucleotides, which codes for the 16S ribosomal RNA that has been suggested to undergo strong adaptive selection for mutations affecting stem-loop secondary structure of the ribosome (50).

In order to estimate selection within genes, we used the more robust comparison between S and NS LONR scores. The significance was estimated using a two sample *t*-test and an FDR correction (51) was applied. In most genes, the difference from the baseline is significant (*P* < 0.001) (dark full and dashed lines in Figure 4). Among the 13 coding regions, there are some prominent areas such as CytB, ND4 where positive selection takes place, and ND2 and COX3 that undergoes negative selection. In the ribosomal RNA, we do not compute an NS to S difference, since we cannot clearly define NS and S mutations. Applying either Tajima's D index or an S to NS measure on the same sequences does not clearly provide a distinction between genes, and does not detect the Ribosomal RNA (Figure 4C). Moreover, Tajima's D and NS fraction detect selection in much less genes.

Specifically, previous measures of selection in mitochondrial genes, using either S to NS mutation ratio (52), relative selective constraint (52,53) or neutrality index (54) are in good agreement with our results, but each of those only produce a sub-set of the results obtained by the LONR. Measures for selection on CytB and Cox3 were consistent with our observation: CytB was consistently found to undergo positive selection (52,53,55). Similarly, COX3 was shown to have relatively low S/NS ratio and high neutrality index (52–55) suggesting a negative selection on this gene. For most genes where we did not discover selection, no stringent selection was reported in the literature. Similarly, as described by (50), mutations are systematically positively selected in the ribosomal RNA. Note that the LONR can provide a very clear estimate of the strength of selection in this region, and it is much stronger than in regular genes.

Still, limited differences exist between some published results and the LONR measure. Mainly that ND4 is claimed to undergo negative selection, and ND2 to undergo positive selection in contrast with our study. ATP6 is reported to pass selection, which is not detected by the in LONR measurement. The source of the difference is probably that we measure relative selection within a population, while NS/S measures are affected by genes and alleles common to the entire population. In other words, traditional measurements estimate whether mutations increase the probability of a cell with a sequence carrying this mutation of being observed, while we measure whether mutations increase the growth rate a sub-population carrying it.

### Viral sequences

**Figure 5.**
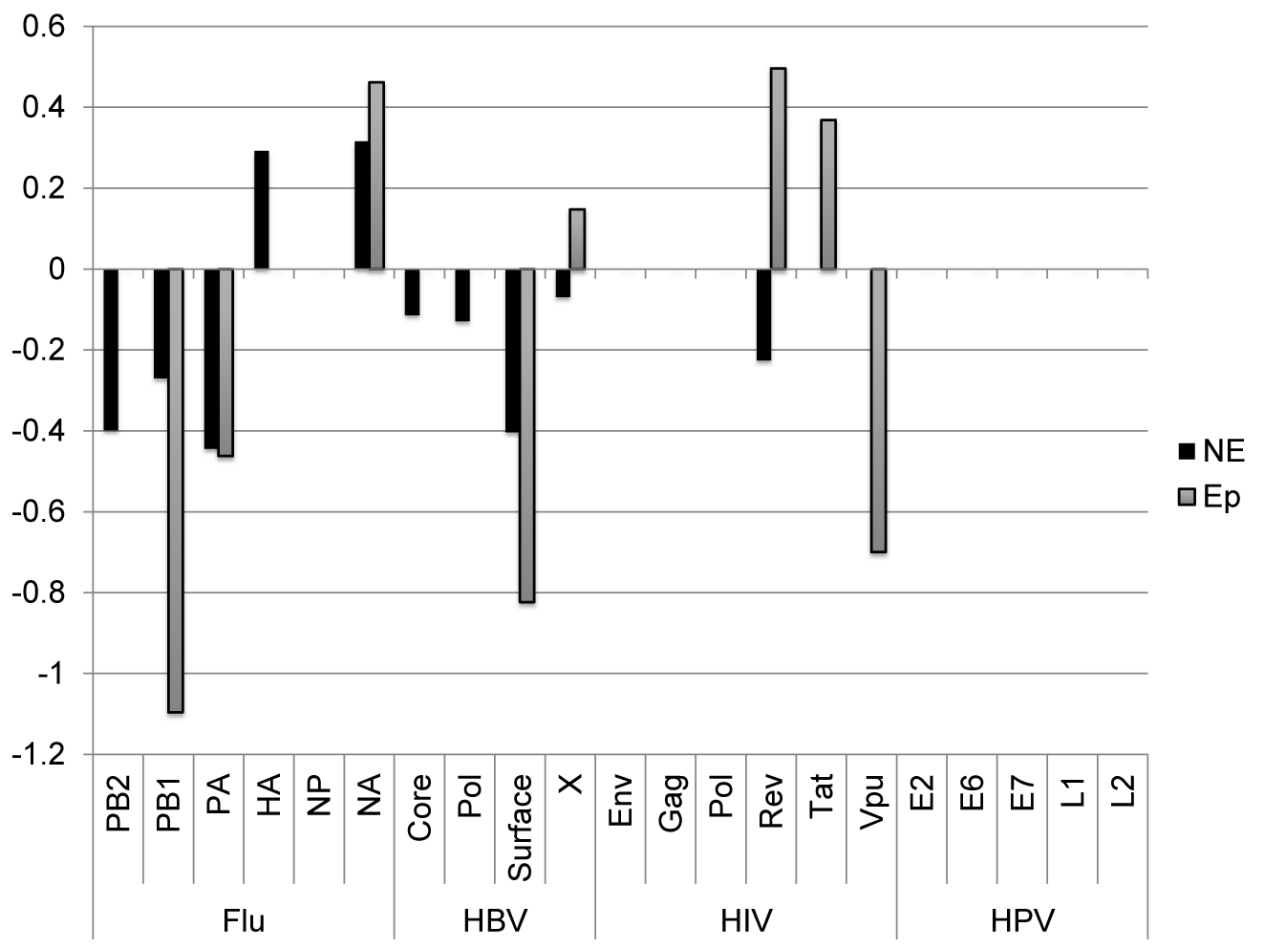
Difference between LONR score for NS and S mutations inside (Ep) and outside (NE) T cell epitopes. Only cases with a *t*-test *P*-value < 0.05 are drawn. All values are given in Table 1. The positive values represent positive selection, and negative values represent negative selection. Positive selection is observed in proteins known to avoid recognition by the immune system.

Another interesting case of population dynamics with a high mutation rate, and an expected strong selection is the escape of viruses from detection by the immune system through mutations in their epitopes. RNA Viruses accumulate mutations at a rate of approximately one mutation per division per genome. We analyzed 21 viral proteins from four organisms (see Materials and Methods), and computed for each protein the difference between the S and NS LONR distributions (Table 1, Figure 5). Proteins were divided into CD8+ T cell epitope and non-epitope regions (see Materials and Methods for a detailed explanation of epitope description).

**Table 1.** Mean LONR NS–S differences for multiple viral proteins, separated into epitope (Ep) and non-epitope (NE) regions

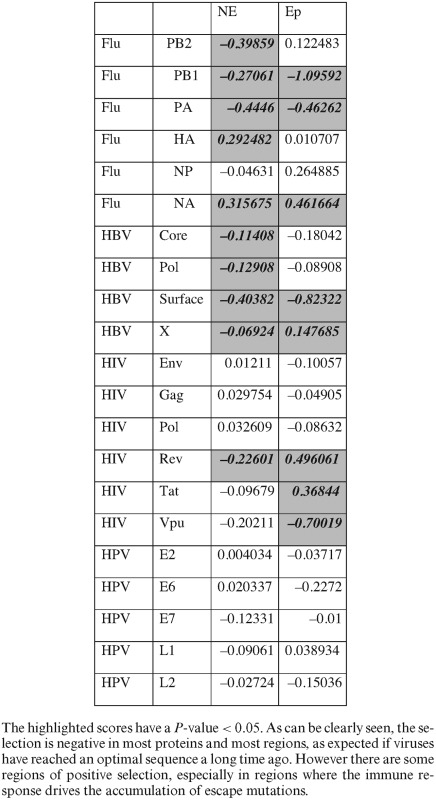			

A strong and significant negative selection was observed in multiple proteins, in both epitope and non-epitope regions (Figure 5) in HIV, Flu and HBV, but not in HPV. Such a selection is expected if viral proteins have reached an optimal sequence a long time ago.

In some proteins, a clear positive selection has been observed in the epitopes (*t*-test *P*-value <0.001 for HIV TAT and HIV Rev) in the two main proteins reported to mutate away their epitopes (40,56–58). Note that this observed selection represents the rapid removal of epitopes and not the removal of epitopes that may have occurred historically, since we only look at mutations that can be computed from the current sequences compared with their Most Recent Common Ancestor (MRCA). Outside T cell epitopes, a positive selection is only observed in the Influenza Hemagglutinin and Neuraminidase, which are known to accumulate mutations to avoid the detection by antibodies and B cells. Thus, the LONR indeed detects the best known targets of positive selection in viruses. Note that it does not detect an advantage for escape mutations in other proteins. There, this advantage may be too weak, or masked by a parallel negative selection, yielding a net unobservable detection.

When using either Tajima's D index or the NS/S measure, systematically, more positive and negative selection are observed outside epitopes than inside epitope (*t*-test on the absolute value of D or [NS/NS+S]-[NS0/NS0+S0] between epitope and non-epitope regions with *P* < 0.001, Supplementary Figure S4). Moreover, negative selection is observed in practically all viral genes, when using the NS/S method (*t*-test of all viral proteins versus 0, *P* < 1.e-4, Supplementary Figure S4). This is probably due to an inaccurate baseline model.

### Mouse immunoglobulin

Probably the most classical real time evolution with a high mutation rate and growth of clones is the affinity maturation process of B cells in germinal centers. In this process, an initial B cell grows into a clone and during its growth hyper-mutations occur in the B cell receptor at an approximate rate of one mutation per division (59), with an extreme division rate (43). They thus fit precisely the LONR framework. We have studied two transgenic mice strains (Lyle and Meg), with pre-defined B cell receptors: one starting with a high affinity receptor to the experimental antigen tested, and one with a low affinity. In order to induce a potent immune response, the low affinity mouse strain must accumulate a large number of specific mutations to obtain a high enough affinity receptor (28). The mouse strain with an initially high affinity can form clones even with the receptor it has, and it is thus intuitively not under a very stringent selection.

Indeed, the initially low affinity mice show a large difference between S and NS mutation LONR scores, with a clear positive selection, while the high affinity mice do not show such a difference (Table 2). Note that in principle these mice could also have a negative selection, where mutations in average reduce the fitness of the cells. However, we do not observe such a selection in these mice strains. Table 2 contains a methodological comparison that will be further explained.

**Table 2.** Mean LONR NS–S differences and two-sample *t*-test values for the two mouse types, calculated using four tree construction algorithms

	NS–S LONR Difference		*P*-value	
	Lyle	Meg	Lyle	Meg
Maximum parsimony	0.6243	0.1926	0.0095	0.2766
Maximum likelihood	0.7475	0.1895	0.0141	0.4502
Neighbor joining	0.5174	0.1945	0.1113	0.5154
UPGMA	−0.3524	−0.0267	0.2813	0.9294

The results are similar for most algorithms, except for the UPGMA, which is quite simplistic and often contains unrealistic assumptions, such as a uniform molecular clock.

### Human immunoglobulin

A more interesting case is the full B cell repertoire of a human host. In such a repertoire, two opposite types of selection operate: (i) mutations can ruin the functionality of the receptor and decrease its survival probability, and (ii) mutations can increase the affinity to the antigen and lead to a higher division rate. The Complementarity Determining Region (CDR) of the B cell receptor determines its interaction with the antigen, and mutations there have a higher probability to increase the affinity than mutations in the framework (FWR) region (8,60). However, the net selection effect in each of these regions still remains unclear. Beyond the effect of somatic hyper-mutation, B cells are affected by isotype switches from naïve IgM to memory IgM, and from there to memory IgG and IgA. The memory (IgM, IgG and IgA) isotypes occur at the advanced stages of the immune response and thus lineage trees based on such receptors are expected to represent the full evolution following selection.

**Figure 6.**
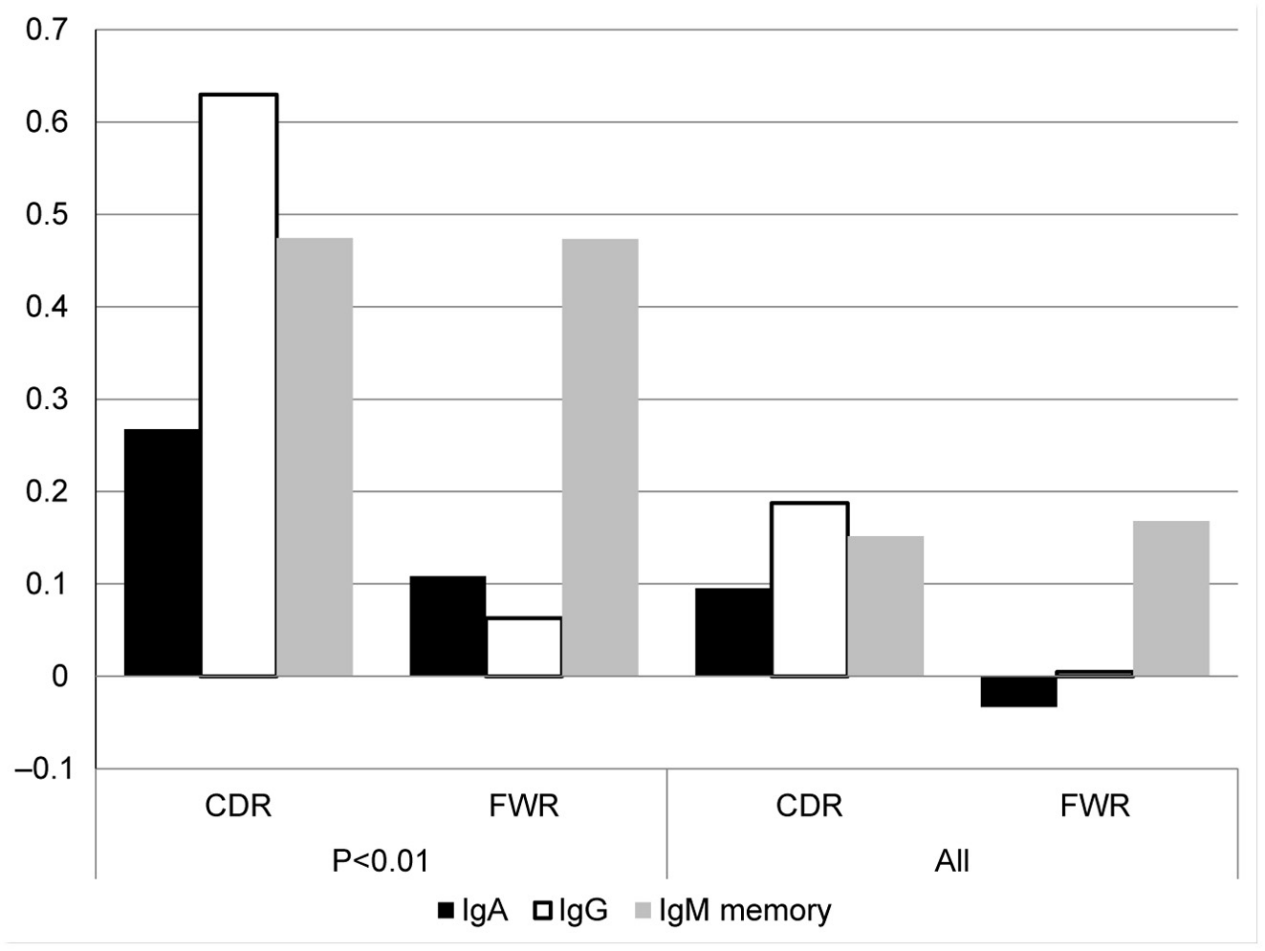
Mean LONR values for immunoglobulin sequence pools. Mean LONR values for memory IgM, IgA and IgG sequence pools, where the values are averaged over (i) all trees in which difference between overall NS and S LONR values was found to be significant (*t*-test, *P* < 0.01) and (ii) all trees.

We have used high-throughput sequencing to sequence over 500 000 B cell receptor samples from each donor, in 12 donors. We built lineage trees from the sequences (see (31) for details of sequences, and production of lineage trees), and measured the LONR distribution in all IgA and IgG sequences trees and compared the LONR distribution in NS and S mutations. At the first stage, we only analyzed trees with significantly different NS and S LONR averages (unpaired two-sided *t*-tests, *P* < 0.01), and analyzed two regions of the B cell receptor where the junctional diversity had no effect on the construction of the lineage trees: FWR3 and CDR2 (61). The results are quite striking. As expected in both IgG and IgA memory cells, the positive selection is much stronger for the CDR region than for the framework (Figure 6). However, even the FWR region passes a positive selection during the immune response. Such a positive selection in the FWR region suggests that the large clones (i.e. clones that were selected to grow more than others), are actually affected by structural changes in the FWR region. This selection may represent the need for structural changes in the immunoglobulin structure to reach a very high affinity.

Interestingly, when analyzing all trees (over 30 000 lineage trees), the reported negative selection in the FWR region (15) appears in the IgA isotypes (Figure 6). This leads to the interesting conclusion that selection may be affecting differently the main part of the distribution and its extremities. In the main part of the distribution, NS mutations in the CDR are selected, since they improve the affinity, and NS mutations in the FWR are selected against since they ruin the structure of the antibody (62). In the extreme cases, the NS mutations in the FWR are also selected, since some of these mutations can actually improve the affinity and enlarge the resulting clones. A much more detailed analysis of this specific data set can be found in (63). The Ig analysis is a classic example of the simultaneous positive and negative selection in different regions of the same gene and of the possibility of detecting such selection using the LONR.

### Effect of tree building algorithm

Constructed lineage trees are only estimates of the real lineage, and their precise shape may be sensitive to the algorithm used to build them and to the baseline mutation model. We have tested whether the methodology used to build the trees affects the LONR scores. We have constructed the lineage trees from the two mouse strains discussed previously using four methods: Maximum Likelihood, Maximum Parsimony, Neighbour Joining and UPGMA. All algorithms were applied using the Phylip toolbox (25). In all methods, except for UPGMA, the LONR results were qualitatively similar (Table 2), with the maximal difference between S and NS mutations being in the MP algorithm. The most significant results were obtained using MP and ML (Lyle results in Table 2). UPGMA is a highly simplistic algorithm and should not be used to detect fine details of tree shapes.

In order to further validate the effect of tree building, we performed simulations where the real tree is known, and computed the LONR on the real tree, instead of the reconstructed tree. We then computed the correlation between the average LONR results in each region using the reconstructed tree and the real trees (*r* > 0.9 (Supplementary Figure S5)).

### Possible confounding effects

When multiple alleles are present in the population, and are integrated into a common lineage tree, the difference between the alleles could be interpreted as selection. A similar effect can occur when a selective sweep occurred in a part of the population, and a large number of mutations are fixated in a sub-population. In principle, this should not affect the LONR, since the selective sweep or the difference between alleles should have a similar effect on S and NS mutations.

Still, we validated that the LONR does not detect a difference between alleles as selection. We simulated populations with two different alleles, where one allele was started with a population twice larger than the other. We then tested for the selection in the mixed population. The results were similar to the results with a single founder allele (Figure 3C and D). We then tested whether removing long branches (branches with more than K mutations) from the analysis would improve the precision of the method. We found no difference between the results with and without long branches, and no selection was observed in regions where selection was not simulated. Thus, the presence of alleles or selective sweeps does not affect our score, and removing long branches (i.e. separating the two alleles) does not improve the score. Still, we leave that as an option for the user.

Another important source of error could be the effect of future mutations on earlier branches. If a mutation drastically increases the fitness of an organism, all mutations occurring on the pathway to the current mutation could be marked as positively selected. Note that this should not in principle affect the LONR based test, since as mentioned above, S and NS mutations would be affected in a similar way. Still, we tested whether the high LONR score in mutations occurring in a given node is only determined by mutations occurring in a given sub-branch. We flagged as tentative mutations in which the LONR is drastically affected by one sub-branch, and tested the selection with and without flagged mutations, and the results were similar (data not shown).

## DISCUSSION

The detection of selection is a crucial issue in population biology, evolution theory and ecology. It also has important clinical implications. While multiple sequence based methods have been proposed to detect selection (15,64–69), most of them are focused on strongly advantageous or deleterious mutations. We have here proposed a method best adapted to the detection of slightly advantageous or deleterious mutations in micro-evolution.

The basic concept behind the here reported LONR measure is to test for the systematic increase of the population size following non-synonymous mutations in a given region. An advantage of the LONR is that each mutation is counted once independently of the total number of sequences that end up containing this mutation. Thus, it is practically unaffected by sampling biases or by the expansion of specific sub-populations.

While multiple tree shape based methods were developed (18–21), these methods often cannot detect the direction of selection, and cannot detect which region in the sequence is selected. Moreover, many of these tree shapes are sensitive to sampling effects making them impractical to use in realistic situations (70).

We have here proposed a new method that can clearly detect positive and negative selection or their combination, based on the effect each mutation has on the number of offspring in the tree under the branch where the mutation has occurred. This method can only be applied where the mutation rate is high enough, and the selection is weak enough for alleles with disadvantageous mutations to exist in the population. Specifically, the mutation rate multiplied by the fixation time of mutations should be much larger than one. The code and a short manual are supplied in the Supplementary Materials.

Such a range exists for example in the population dynamics of mitochondria within host species, in viral dynamics and in the affinity maturation process in germinal centers. We have here studied all these cases and have shown that indeed selection can be detected in all cases studied. Other applications of this method can be the evolution of the Y chromosome and the changes in Short Tandem Repeats (STR) frequencies in it or the evolution of bacteria in an infection in the population.

This method is precise in the domain of a large number of mutations per sequence (>10) and large samples (>300). In this domain, the method proved to have many important applications, such as the detection of selection in genes (and actually the direct detection of genes), the detection of viral proteins passing positive and negative selection and understanding the selection process in a B cell immune response. We have shown that while the ribosomal RNA has a very strong positive selection, some genes pass positive selection, and others negative selection. In B cells, we have shown that while CDR mutations are always positively selected, FWR mutations are selected against in the majority of the populations, but actually strongly positively selected in the extreme cases.

The comparison between S and NS mutations is only the most basic distinction between different types of mutations. Other possibilities exist, especially change/no-change of some amino-acid property, such as size or hydrophobicity. Such methods would test for selection for specific changes and not selection for mutation in general. In other words, the proposed methodology can be used to estimate whether changes in a given property increase or decrease the number of offspring, compared with a change not altering this properties. In other words, different definition of the mutations of interest and the baseline can be defined, and used to detect selection for other features.

The main limitation of the current score is that it is blind to strong selection. Once a mutation is fixed in (or completely removed from) the population, we will not observe the polymorphism at this site that allows us to compare the branch sizes.

## Supplementary Material

SUPPLEMENTARY DATA
