# Supplementary material for: Estimate of within population incremental selection through branch imbalance in lineage trees
Source: Nucleic Acids Res. 2015 Nov 19;44(5):e46. doi: 10.1093/nar/gkv1198 (PMC4797263; doi:10.1093/nar/gkv1198)
Supplement: SUPPLEMENTARY DATA [file supp_44_5_e46__index.html]

Estimate of within population incremental selection through branch imbalance in lineage trees — Estimate of within population incremental selection through branch imbalance in lineage trees — SUPPLEMENTARY DATA 

# Estimate of within population incremental selection through branch imbalance in lineage trees

## SUPPLEMENTARY DATA

- SUPPLEMENTARY DATA
- SUPPLEMENTARY DATA
