# Supplementary material for: Estimate of within population incremental selection through branch imbalance in lineage trees
Source: Nucleic Acids Res. 2015 Nov 19;44(5):e46. doi: 10.1093/nar/gkv1198 (PMC4797263; doi:10.1093/nar/gkv1198)
Supplement: SUPPLEMENTARY DATA [file supp_gkv1198_nar-01244-met-n-2015-File010.docx]

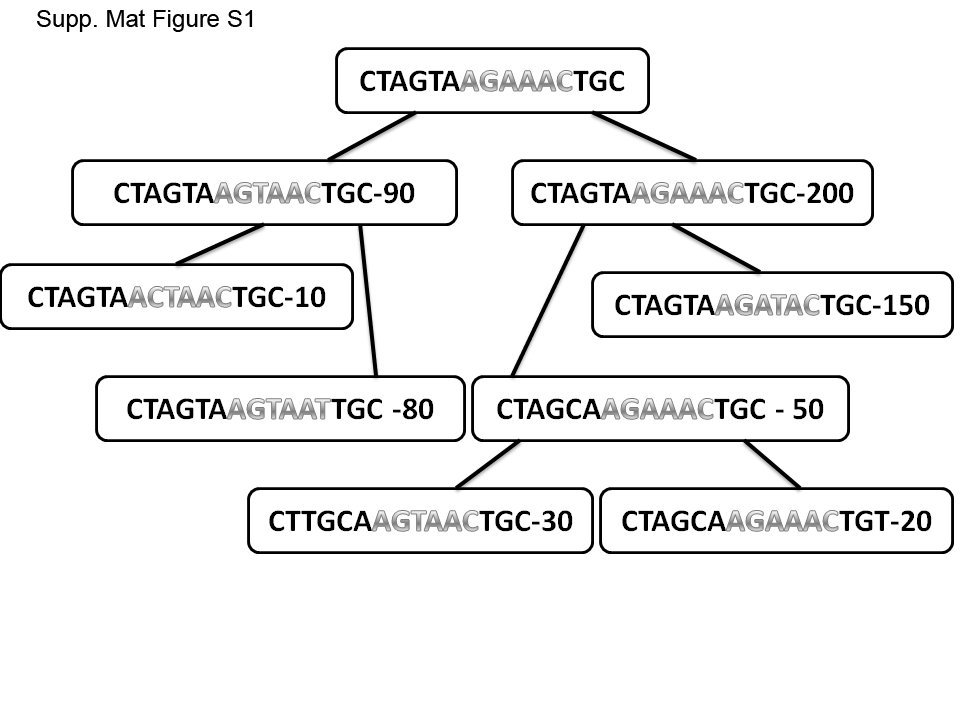
**Supplementary Figure 1.** Typical tree with the sequences in each node and the total number of offspring of each node. Only internal nodes are drawn here (leaves are not used for the analysis, since they have no mutations under them). The sequences are divided into two regions (different colors). The number in each node is the total number of offspring.

**Supplementary Figure 2.**  LONR values for S and NS mutations as given in Supplementary Figure 1. For each mutation its region is give (1 or 2), its type (S or NS), its position along the gene in nucleotides, the mutation itself in nucleotides and amino acids, as well as the LONR score. The distribution of LONR scores is used to asses selection.


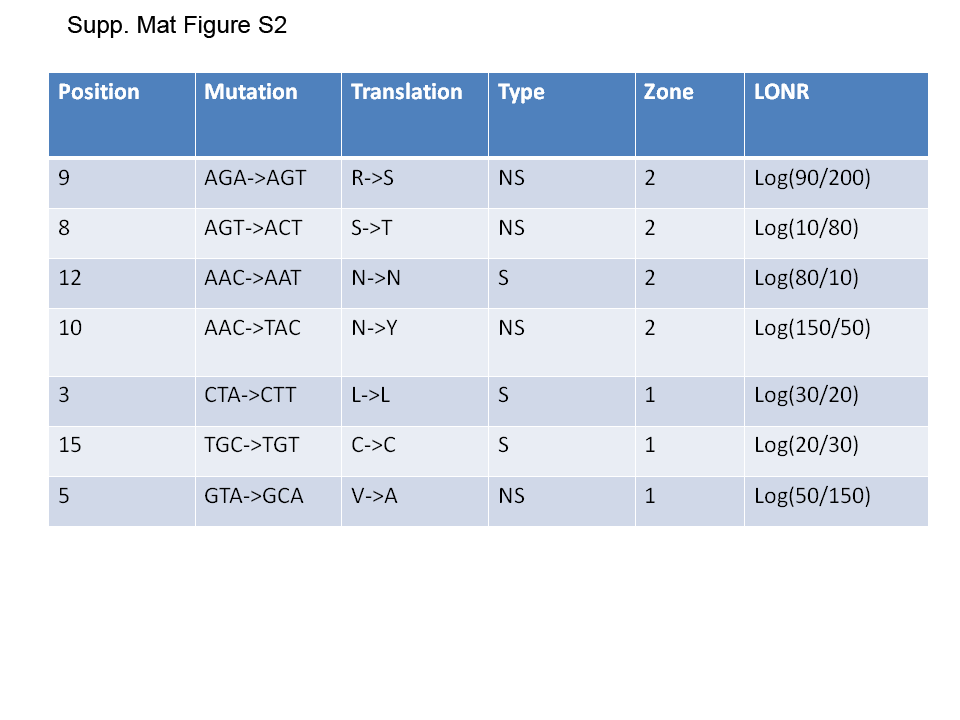


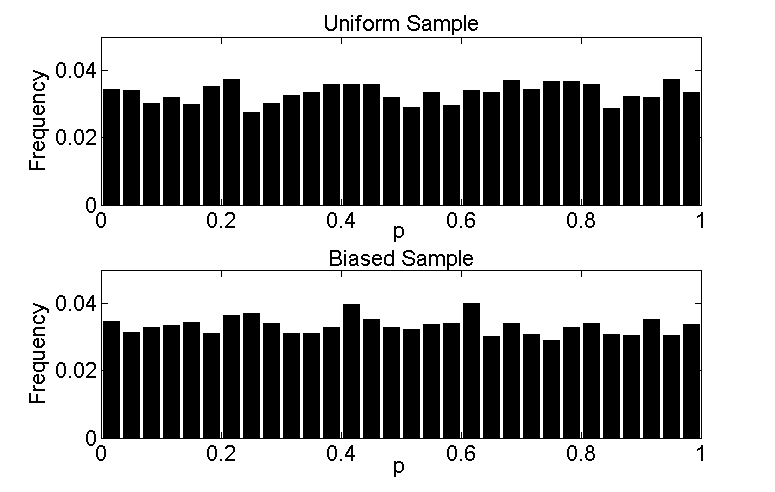


**Supplementary Figure 3.** Distribution of P values from LONR estimate on a coalescent simulation. A coalescent was built on a population of 100-10,000 samples. A randomly chosen sequence was assigned as the root of the coalescent, and different mutation models and rates were tested. The distribution of p values was computed over all realizations. One can clearly see (upper plot) that the distribution is uniform over all values between 0 and 1, as expected from a neutral model. We then repeated the analysis when a branch containing half the leaves was sampled at half the frequency of the other branch. The results are similar (lower plot). The results were not affected by the mutation rate or the mutation model.

**Supplementary Figure 4.** Tajima’s D and NS/(NS+S)-NS0/(NS0+S0) scores for mutations inside (Ep) and outside (NE) T cell epitopes. The positive values represent positive selection, and negative values represent negative selection. Positive selection is observed in proteins known to avoid recognition by the immune system. The relative mutation probabilities were computed using a model with a twice higher transition than transversion probability. The upper plot is Tajima D index, and the lower plot is the NS/(S+NS) ratio.


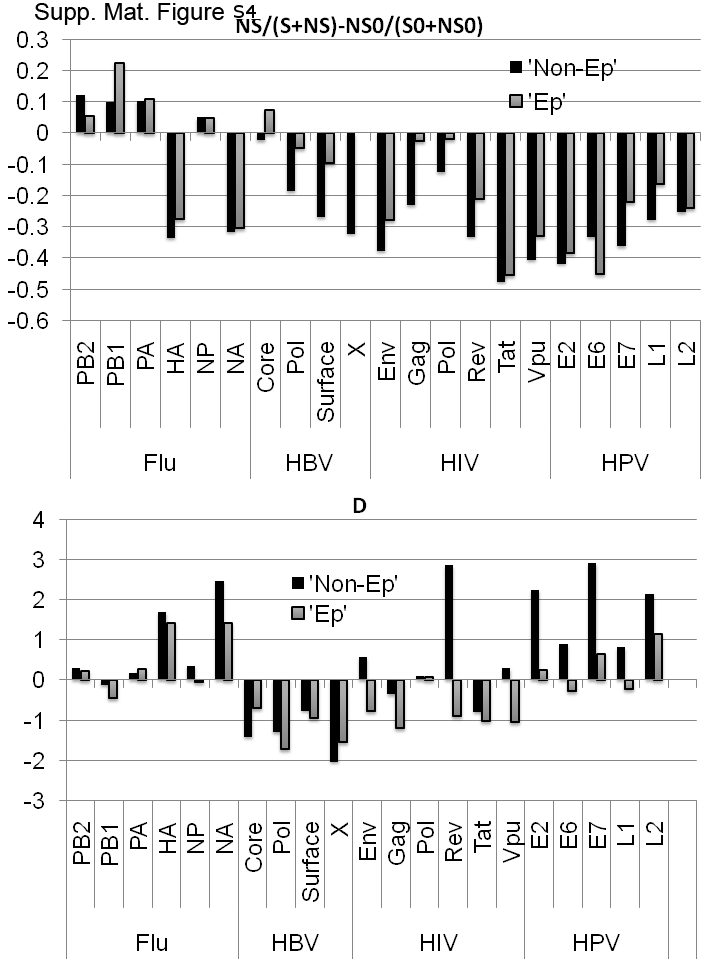


**Supplementary Figure 5.** Average LONR score per region using 20 simulated trees. The x axis is the value computed assuming the real lineage tree is known, and the y axis is from a NJ based reproduction of the trees. The line is the x=y lines. One can see that in all regions, there is a good agreement between the results obtained with the real and reconstructed trees.


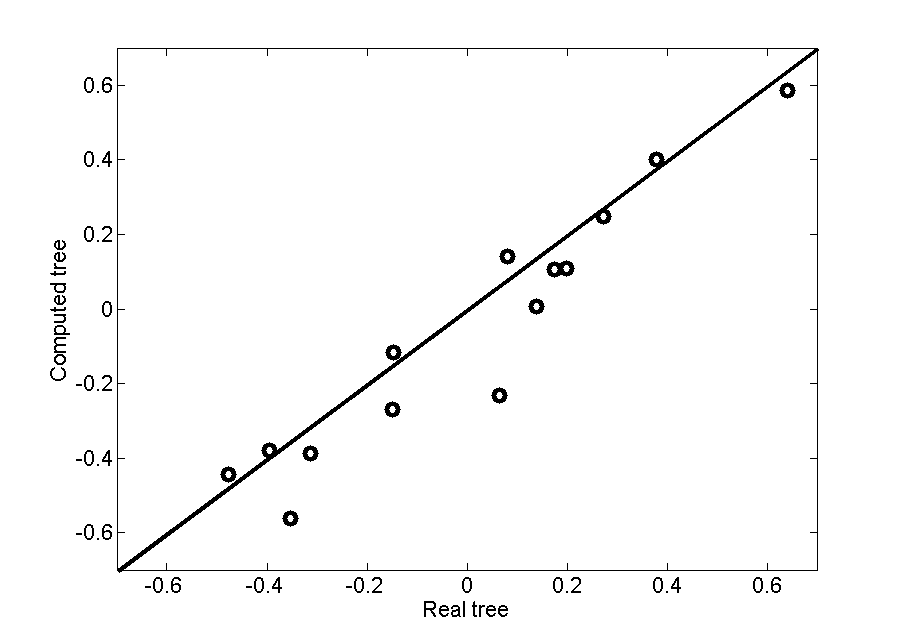


# Computing LONR scores in lineage trees

## General

The program is written in **R**.

This analysis is divided into two parts:

First, a lineage tree is built with sequences provided in and **aligned FASTA format**. There is an option to provide an outgroup sequence in order to detect more accurately the root sequence. For less than 100 sequences, the Maximum parsimony method is used (*dnapars* Phylip program). Otherwise, the Neighbor-joining method is used (*neighbor* Phylip program). The Neighbor-joining method uses a distance matrix as input, which is computed using the *dnadist* Phylip program. The internal sequences are reconstructed using the Fitch algorithm. The tree is then divided into subtrees in order to ignore mutations occurring in very distant sequences. Thus the user can specify the cut-off (in nucleotides) to cut long branches. The default is 10 mutations. The following LONR analysis is then performed for each subtree separately.

Second, mutations are detected between each pair of father and son sequences (at the nucleotide level). The LONR score is calculated, for each mutation, as the log of the ratio between the sub-tree size of the son in which the mutation occurred and the sub-tree size of the son in which no mutation occurred at this position.

## How to run

This program uses the ***Phylip-3.695*** package. It is expecting it to be in the same folder as this script. The path to the program can be modified in the beginning of the script.

The package ***Biostrings*** and ***seqinr*** need to be installed.

Main function *-* ***compute.LONR()***

## Input

1. *in.dir* – aligned FASTA file input directory
2. *out.dir* – output directory for tree files and LONR results
3. *file* – FASTA file name
4. *outgroup* (*optional*) – outgroup sequence name. If
5. *cutoff* - branches with more mutations than specified nu this cutoff will be trimmed, resulting in several subtrees (default - 10 )

## Output

The following directories are created in *out.dir*:

*Tree* directory –

1. *filename*.fasta -modified FASTA file if gaps were removed.
2. *filename*.dis – (only for neighbor joining trees) contains distance matrix created by the *dnadist* program in the Phylip package.
3. *filename.*phy – original sequences in alignment format.
4. *filename*_edges.tab – tab-delimited file containing the tree edges, their weights and the distance in nucleotides between each two nodes.
5. *filename*_names.tab – tab-delimited file matching between original sequence names and temporary names.
6. *filename*_out.txt – Phylip output tree file
7. *filename*_tree.txt – Phylip output tree file in Newick format

*LONR* directory –

1. *filename*_lonr.csv – comma-separated file containing lonr results as followed:
2. *mutation –* mutated nucleotides (e.g. AC means A🡪 C)
3. *LONR –* log(size of mutated sub-tree/size of un-mutated sub-tree)
4. *mutation.type –* (S) Silent or (Replacement)
5. *position* – in nucleotides, according to output FASTA file (see above)
6. *father* – sequence name **from** which occurred mutation
7. *son* – sequence name **to** which occurred mutation
8. *flag* – True if LONR score an internal node is affected by mutations occurring in its descendants

## Clarifications

1. The input sequences must already be aligned. If there are gaps, the consensus sequence of all the sequences is computed, and positions containing gaps are removed from all the sequences. The output FASTA file is created after this step.
2. The *dnapars* program may create trees which are not completely binary. Thus, internal nodes which have more than two children are fixed by created an identical new child, which will receive the extra children.

This also happens is case no outgroup is provided and the root has three children (both in *dnapars* and *neighbor* programs).

1. If the nucleotide sequence lengths are not a multiple of three and mutations occurred in the last nucleotides, these mutations are ignored since they cannot be typed (not a full codon).
